# Potassium responses to sodium zirconium cyclosilicate in hyperkalemic hemodialysis patients: post-hoc analysis of DIALIZE

**DOI:** 10.1186/s12882-021-02569-7

**Published:** 2022-02-08

**Authors:** Steven Fishbane, Martin Ford, Masafumi Fukagawa, Kieran McCafferty, Anjay Rastogi, Bruce Spinowitz, Konstantin Staroselskiy, Konstantin Vishnevskiy, Vera Lisovskaja, Ayman Al-Shurbaji, Nicolas Guzman, Sunil Bhandari

**Affiliations:** 1 Department of Medicine, Zucker School of Medicine at Hofstra/Northwell, 100 Community Dr, Great Neck, NY 11021 USA; 2 Department of Renal Medicine, King’s College Hospital NHS Trust, London, UK; 3 Faculty of Life Sciences and Medicine, King’s College, London, UK; 4 Division of Nephrology, Endocrinology and Metabolism, Department of Internal Medicine, Tokai University School of Medicine, Isehara, Japan; 5 Department of Nephrology, Bart’s Health NHS Trust, London, UK; 6 UCLA CORE Kidney Program, University of California Los Angeles, Los Angeles, CA USA; 7 Department of Medicine, New York-Presbyterian Queens, Queens, NY USA; 8 Department #2, B. Braun Avitum Russland Clinics, St Petersburg, Russia; 9 Propedeutics of Internal Diseases Chair, First Pavlov State Medical University of St Petersburg, St Petersburg, Russia; 10 Biometrics and Information, AstraZeneca BioPharmaceuticals R&D Gothenburg, Mölndal, Sweden; 11 Global Medicines Development, AstraZeneca BioPharmaceuticals R&D Gothenburg, Mölndal, Sweden; 12 Global Medicines Development, AstraZeneca BioPharmaceuticals R&D, Gaithersburg, MD USA; 13 Department of Renal and Transplant Medicine, Hull University Teaching Hospitals NHS Trust, Hull, UK

**Keywords:** Hemodialysis, Chronic kidney disease, Potassium, Hyperkalemia, Sodium zirconium cyclosilicate

## Abstract

**Background:**

Sodium zirconium cyclosilicate (SZC) is an effective and well-tolerated treatment for hyperkalemia in maintenance hemodialysis patients. In post-hoc analyses of the phase 3b DIALIZE study, we examined the spectrum of potassium responses to SZC.

**Methods:**

Post-hoc analyses with SZC and placebo included: the number of long interdialytic interval (LIDI) visits during the 4-week evaluation period where patients attained pre-dialysis serum potassium (sK^+^) concentrations of 4.0–5.0 and 4.0–5.5 mmol/L; potassium gradient (the difference between pre-dialysis sK^+^ and dialysate potassium) at days 36, 43, 50, and 57, and change from baseline to the end of treatment (EOT) using categories of potassium gradient (1 to < 2, 2 to < 3, 3 to < 4, and ≥ 4 mmol/L).

**Results:**

A greater proportion of patients achieved the ranges of pre-dialysis sK^+^ concentration with SZC versus placebo for ≥1, ≥ 2, ≥ 3, and 4 LIDI visits over 4 weeks; 23.7 and 48.5% of patients in the SZC group achieved pre-dialysis sK^+^ concentrations of 4.0–5.0 and 4.0–5.5 mmol/L, respectively, at all 4 LIDI visits. Baseline mean potassium gradient was similar with SZC and placebo. At day 57, mean (standard deviation) potassium gradient was 2.78 (0.08) mmol/L with SZC and 3.52 (0.08) mmol/L with placebo; mean difference (95% confidence interval) was − 0.74 mmol/L (− 0.97 to − 0.52). A greater reduction in potassium gradient category from baseline towards lower-risk categories at EOT was observed with SZC versus placebo.

**Conclusions:**

These analyses expand our knowledge of the spectrum of potassium responses with SZC in hyperkalemic hemodialysis patients.

**Trial registration:**

NCT03303521.

## Background

Patients with end-stage kidney disease (ESKD) have severely reduced renal potassium excretion. Excessively high serum potassium (sK^+^) is known to promote ventricular arrhythmias and cardiac death; however, even modest increases, such as > 5.5 mmol/L, are associated with increased all-cause mortality and hospitalization [1–4].

Patients with ESKD require dialysis to remove accumulated potassium, with a goal of maintaining or restoring sK^+^ to within the normal range. Maintenance hemodialysis is typically performed three times weekly, with two 1-day intervals (i.e., the short interdialytic interval [SIDI]) and one 2-day interval (the long interdialytic interval [LIDI]) between dialysis sessions [5]. During dialysis, potassium has the potential to move freely across the dialyzer membrane, typically down a concentration gradient from a patient’s blood into the dialysate [6]. Therefore, dialysate potassium (dK^+^) concentration is a modifiable factor that affects the sK^+^ concentration achieved during hemodialysis [7, 8]. Studies to date on the impact of low dK^+^ baths (typically defined as 0–1 mmol/L) on hemodialysis outcomes have yielded contrasting results. Some studies have suggested no increased risk with low dK^+^ baths [1, 9], while others have reported an increased risk of mortality, sudden death, or sudden cardiac arrest [4, 10–13].

The potassium gradient results from the difference between a patient’s sK^+^ and dK^+^ concentrations. A low gradient among patients with high sK^+^ may result in insufficient potassium removal during dialysis, which is associated with a greater risk of hyperkalemia and increased mortality [1, 4, 13]. Dialysis with a high gradient, resulting from a high sK^+^ and/or low dK^+^, leads to greater removal of potassium than with a lower gradient [7] and has utility in controlling hyperkalemia among patients with high sK^+^ [1]. However, a high potassium gradient at the start of hemodialysis also causes more rapid fluxes of potassium and is associated with a greater risk of adverse events (AEs), such as cardiac arrhythmia, mortality, and hospitalization [1, 13–16]. Therefore, physicians face a challenge to balance the need to remove sufficient potassium to avoid hyperkalemia, while minimizing the risk of AEs.

Hemodialysis patients with hyperkalemia often depend on additional strategies to manage hyperkalemia, typically including dietary counseling, therapies such as potassium binders or, in more extreme situations, longer or more frequent dialysis. Sodium zirconium cyclosilicate (SZC) is a novel, highly selective potassium binder that is not absorbed or metabolized by the body [17]. SZC preferentially captures potassium in the gastrointestinal lumen, thereby reducing potassium absorption and increasing potassium fecal excretion, and reducing sK^+^ [17–19]. The efficacy and safety of SZC for the treatment of hyperkalemia have been demonstrated in phase 2 and 3 studies of non-dialysis populations with chronic kidney disease, heart failure, and diabetes [18–23], as well as in hemodialysis patients [24]. The phase 3b DIALIZE study demonstrated that SZC is an effective and well-tolerated treatment for hyperkalemia in patients with ESKD undergoing maintenance hemodialysis [24]. Overall, 41.2% of patients receiving SZC and 1.0% of patients receiving placebo were deemed to be treatment responders [24].

Here, we report the results of post-hoc analyses of the DIALIZE data performed to further examine the spectrum of potassium responses to SZC, in terms of sK^+^ control and potassium gradient, in hyperkalemic hemodialysis patients.

## Methods

### Study design and patients

The full details of the DIALIZE study (NCT03303521) have been presented previously [24]. Briefly, DIALIZE was a randomized, double-blind, placebo-controlled, phase 3b study conducted across Japan, Russia, the US, and the UK. The study consisted of a 1-week screening period, an 8-week treatment period comprising 4 weeks for SZC dose titration and 4 weeks for evaluation on stable dose, and a 2-week follow-up period. Patients were randomized 1:1 to receive orally a starting dose of 5 g of SZC or placebo once daily on non-dialysis days (4 days/week). All patients received hemodialysis three times weekly and routine dietary counseling as per local guidelines, including dietary potassium restriction. During dose titration, doses of SZC and placebo were adjusted weekly over 4 weeks to achieve and maintain a pre-dialysis sK^+^ concentration of 4.0–5.0 mmol/L after the LIDI. Doses of SZC and placebo were titrated in 5 g increments to a maximum dose of 15 g once daily on non-dialysis days (4 days/week).

Eligible patients were adults aged ≥18 years with ESKD who were managed for ≥3 months before randomization by hemodialysis three times weekly. During screening, patients were required to have hyperkalemia despite maintenance hemodialysis, defined as pre-dialysis sK^+^ > 5.4 mmol/L after the LIDI on day − 7 and pre-dialysis sK^+^ > 5.0 mmol/L after ≥1 SIDI on days − 5 and − 3.

### Procedures

Samples of sK^+^ were measured using central laboratory assessment and a point-of-care i-STAT device (Abbott Point of Care, Inc., Princeton, NJ, USA). All dose adjustments were based on pre-dialysis sK^+^ measured by i-STAT. Central laboratory samples were obtained throughout the study (both pre- and post-dialysis at LIDI visits, and only pre-dialysis at SIDI visits).

Prescription of dK^+^ was recorded at randomization, during dose titration and at weekly intervals during the evaluation period. For pre-dialysis sK^+^ concentrations < 4.0 mmol/L, subsequent adjustments in dK^+^ concentration could be made according to locally accepted clinical practice patterns guided by the investigator’s clinical judgment. For centers that modified dK^+^ concentration when pre-dialysis sK^+^ concentration decreased, dK^+^ concentration could be increased by 0.5 or 1.0 mmol/L if pre-dialysis sK^+^ was < 4.0 mmol/L. If dK^+^ concentration could no longer be increased during the treatment period (e.g. the dK^+^ concentration was 4.0 mmol/L), the dose of SZC could be decreased by 5 g or held if the patient was already taking the minimum dose (5 g). For sites where local clinical practice did not include increasing the dK^+^ concentration when pre-dialysis sK^+^ fell, the dose of SZC or placebo could be decreased by 5 g or held if the patient was already taking the minimum dose.

### Post-hoc analysis

In these post-hoc analyses, we assessed the number of LIDI visits during the evaluation period (days 36, 43, 50, 57) where patients attained pre-dialysis sK^+^ ranges with SZC and placebo. Pre-dialysis sK^+^ ranges analyzed were 4.0–5.0 mmol/L and an extended range of 4.0–5.5 mmol/L; the latter reflected a range deemed to be acceptable in clinical practice, as sK^+^ concentrations > 5.5 mmol/L are associated with increased hospitalization and mortality [1, 2, 4]. Furthermore, we assessed the potassium gradient at days 36, 43, 50, and 57 with SZC and placebo, as well as the change from baseline to the end of the evaluation period (day 57) using a categorization of potassium gradient (1 to < 2, 2 to < 3, 3 to < 4, and ≥ 4 mmol/L). A potassium gradient of 2 to < 3 mmol/L was determined to be of lower risk, based on a recent report where this range was used as the referent potassium gradient category; categories above this were associated with hospitalization and emergency department visits [16].

### Statistical analysis

All statistical analyses were performed using SAS v9.4 (SAS Institute, Cary, NC, USA). The number of LIDI visits during the evaluation period where patients achieved sK^+^ ranges (4.0–5.0 or 4.0–5.5 mmol/L) was summarized descriptively using percentages. Patients receiving rescue therapy were included in the analysis. No imputation of missing data was conducted. For potassium gradient, at each visit all estimates and 95% confidence intervals (CIs) in the evaluation period were obtained from a linear model with gradient as response and treatment as the single covariate. For days 36, 43, 50, and 57, the mean and standard deviation (SD) for each treatment group are the least-squares mean from this model, which was fitted for each visit separately. For change from baseline to the end of the evaluation period in categories of potassium gradient, baseline was defined as visit 1 (day − 7), and percentages were calculated using the number of patients with an available baseline value.

## Results

### Patients

In DIALIZE, 97 and 99 patients were randomized to receive SZC and placebo, respectively. At baseline, 58.7% of patients were men, mean (SD) age was 58.1 (13.7) years, and mean (SD) weight was 71.0 (19.1) kg (Table 1). Most patients were White (52.0%), Asian (33.7%), or black or African American (9.7%; Table 1).

**Table 1:** Baseline patient characteristics

Characteristic	SZC (***n*** = 97)	Placebo (***n*** = 99)
Age, years, mean (SD)	55.7 (13.8)	60.4 (13.2)
Sex, male, *n* (%)	57 (58.8)	58 (58.6)
Race, *n* (%)		
White	50 (51.5)	52 (52.5)
Black or African American	11 (11.3)	8 (8.1)
Asian	33 (34.0)	33 (33.3)
American Indian or Alaska Native	1 (1.0)	2 (2.0)
Other	2 (2.1)	4 (4.0)
Height, cm, mean (SD)	166.4 (9.9)	165.1 (9.2)
Weight, kg, mean (SD)	72.0 (22.0)	70.0 (15.9)
BMI, kg/m^2^, mean (SD)	26.9 (7.1)	26.7 (5.4)
Pre-dialysis serum potassium concentration, mmol/L, mean (SD)^a^	5.8 (0.6)	5.9 (0.6)
**Dialysis history**		
Vintage, years, mean (SD)	8.0 (6.1)	7.8 (7.6)
Access type, *n* (%)		
Arteriovenous fistula	84 (87.5)	90 (90.9)
Arteriovenous graft	7 (7.3)	3 (3.0)
Tunneled central venous catheter	4 (4.2)	6 (6.1)
Other	1 (1.0)	0 (0.0)
Total	96 (100.0)	99 (100.0)
**Dialysis adequacy**		
spKt/V, mean (SD)	1.7 (0.3)	1.7 (0.4)
Urea removal rate, %, mean (SD)	72.9 (6.7)	74.6 (5.6)
Dialysate flow, ml/min, mean (SD)	512.0 (162.8)	538.5 (136.0)
Dialysis potassium concentration, mmol/L		
Mean (SD)	2.26 (0.49)	2.26 (0.47)
Minimum, maximum	1.0 to 3.0	1.0 to 3.0
Blood flow, ml/min, mean (SD)	322.0 (110.7)	318.5 (96.3)

Republished with the permission of American Society of Nephrology, from A Phase 3b, Randomized, Double-Blind, Placebo-Controlled Study of Sodium Zirconium Cyclosilicate for Reducing the Incidence of Predialysis Hyperkalemia, Fishbane S et al. *JASN*. Sep 2019;30(9):1723-1733; permission conveyed through Copyright Clearance Center, Inc.

*BMI* body mass index, *SD* standard deviation, *spKt/V* single-pool Kt/V, *SZC* sodium zirconium cyclosilicate

^a^Visit 4 (Day 1)

### Control of hyperkalemia

**Fig. 1:**
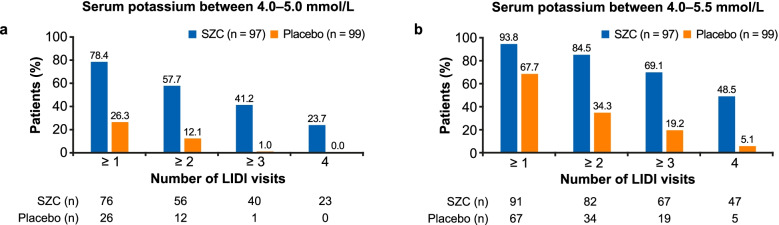
Proportion of patients achieving pre-dialysis serum potassium ranges of **a** 4.0–5.0 mmol/L and **b** 4.0–5.5 mmol/L for ≥1, ≥ 2, ≥ 3, and 4 LIDI visits during the 4-week evaluation period. Includes pre-dialysis serum potassium values obtained at the LIDI visits in the evaluation period (days 36, 43, 50, and 57). No imputation of missing data was conducted. Patients receiving rescue therapy were included in the analysis. LIDI, long interdialytic interval; SZC, sodium zirconium cyclosilicate

Baseline mean (SD) pre-dialysis sK^+^ was comparable for SZC and placebo: 5.8 (0.6) mmol/L versus 5.9 (0.6) mmol/L, respectively (Table 1). SZC was associated with a greater proportion of patients achieving pre-dialysis sK^+^ of 4.0–5.0 mmol/L versus placebo for ≥1, ≥ 2, ≥ 3, and 4 LIDI visits during the evaluation period (Fig. 1a). Overall, 78.4% of patients receiving SZC achieved pre-dialysis sK^+^ of 4.0–5.0 mmol/L at 1 of the 4 LIDIs during the evaluation period, versus 26.3% of patients receiving placebo (Fig. 1a). Furthermore, 41.2 and 23.7% of patients receiving SZC achieved pre-dialysis sK^+^ of 4.0–5.0 mmol/L at 3 out of 4 and all 4 LIDI visits, respectively, versus 1.0 and 0% of patients receiving placebo, respectively (Fig. 1a). Similar findings were observed using the extended pre-dialysis sK^+^ range of 4.0–5.5 mmol/L, although greater proportions of patients achieved the range (Fig. 1b). Overall, 69.1 and 48.5% of patients receiving SZC achieved pre-dialysis sK^+^ of 4.0–5.5 mmol/L at 3 out of 4 and all 4 LIDI visits, respectively, versus 19.2 and 5.1% of patients receiving placebo, respectively (Fig. 1b).

### Dialysate potassium

Baseline mean (SD) dK^+^ concentration was comparable for SZC and placebo: 2.26 (0.49) mmol/L (minimum, maximum: 1.0 to 3.0) versus 2.26 (0.47) mmol/L (minimum, maximum: 1.0 to 3.0), respectively (Table 1). Despite significantly reduced pre-dialysis sK^+^, mean dK^+^ concentration was largely unchanged at the end of treatment (EOT). At day 57, mean (SD) dK^+^ concentration with SZC was 2.28 (0.47) mmol/L (minimum, maximum: 1.0 to 3.0) and with placebo was 2.25 (0.48) mmol/L (minimum, maximum: 1.0 to 3.0). Concentration of dK^+^ was increased in only 2 (2.3%) patients receiving SZC and 1 (1.1%) patient receiving placebo.

### Potassium gradient

**Fig. 2:**
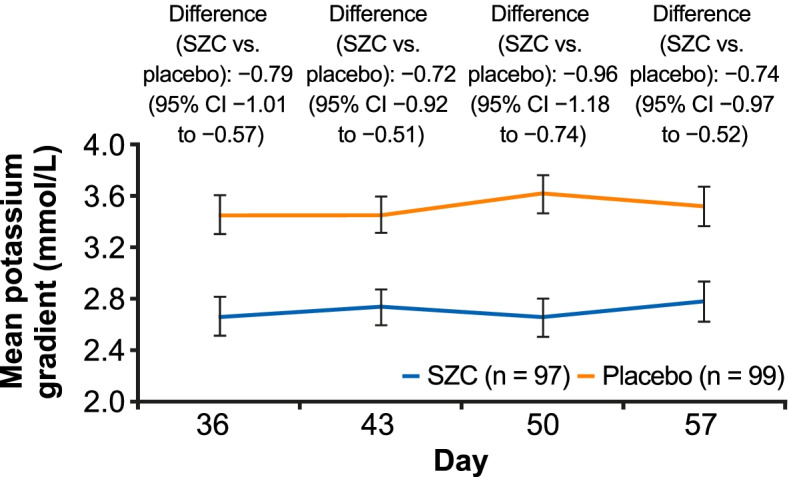
Mean serum potassium to dialysate potassium gradient (mmol/L) during the 4-week evaluation period. Baseline (visit 1, day −7) mean (SD) potassium gradient was comparable between the treatment arms: SZC 3.78 (0.59) mmol/L versus placebo 3.73 (0.64) mmol/L. Error bars represent 95% CIs. All estimates and 95% CIs in the 4-week evaluation period are obtained from a linear model with gradient as response and treatment as the single covariate. The mean for each treatment group is the least-squares mean from this model. The model was fitted for each visit separately. CI, confidence interval; SD, standard deviation; SZC, sodium zirconium cyclosilicate

Baseline (visit 1, day − 7) mean (SD) potassium gradient was comparable for SZC and placebo: 3.78 (0.59) mmol/L versus 3.73 (0.64) mmol/L, respectively. During the evaluation period, potassium gradient was lower with SZC versus placebo at each of the 4 LIDI visits (Fig. 2). At day 57, mean (SD) potassium gradient was 2.78 (0.08) mmol/L with SZC and 3.52 (0.08) mmol/L with placebo; mean difference of − 0.74 mmol/L (95% CI − 0.97 to − 0.52; Fig. 2).

**Fig. 3:**
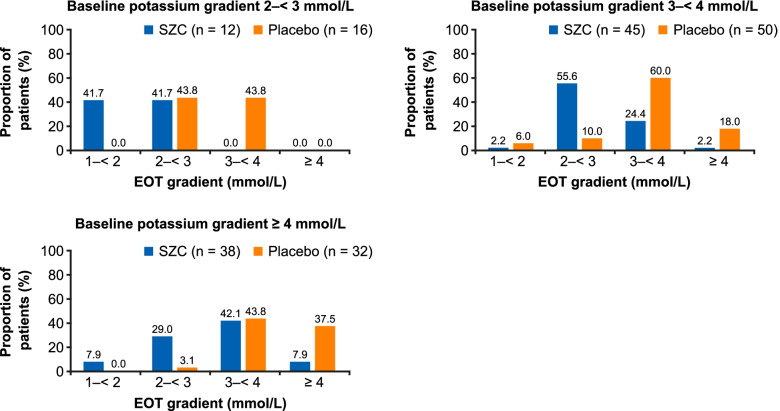
Change in serum potassium to dialysate potassium gradient categories from baseline to EOT with SZC and placebo. Missing data are not shown. Dialysate potassium gradient data at EOT are missing for the following baseline categories: baseline gradient 2–< 3 mmol/L: SZC *n =* 2 (16.7%), placebo *n =* 2 (12.5%); baseline gradient 3–< 4 mmol/L: SZC *n =* 7 (15.6%), placebo *n =* 3 (6.0%); baseline gradient ≥4 mmol/L: SZC *n =* 5 (13.2%), placebo *n =* 5 (15.6%). EOT, end of treatment; SZC, sodium zirconium cyclosilicate

At baseline, 12, 45, and 38 patients receiving SZC had potassium gradients of 2 to < 3, 3 to < 4, and ≥ 4 mmol/L, respectively. In the placebo group, 16, 50, and 32 patients had baseline potassium gradients of 2 to < 3, 3 to < 4, and ≥ 4 mmol/L, respectively. No patient in either treatment group had a baseline potassium gradient of 1 to < 2 mmol/L. A greater reduction in potassium gradient categories from baseline to EOT (day 57) was observed with SZC than with placebo (Fig. 3); furthermore, the magnitude of reduction was dependent on baseline potassium gradient (Fig. 3). With SZC, 55.6% (*n =* 25/45) and 29.0% (*n =* 11/38) of patients with baseline potassium gradients of 3 to < 4 and ≥ 4 mmol/L, respectively, had reductions to a lower risk gradient category of 2–3 mmol/L (Fig. 3). This compared with 10% (*n =* 5/50) and 3.1% (*n =* 1/32), respectively, of patients receiving placebo (Fig. 3), suggestive of regression to the mean in these patients with a higher baseline potassium gradient. In the SZC group, no patients with a baseline potassium gradient of 2 to < 3 mmol/L (*n =* 12) had an increase in gradient to a higher risk category; 41.7% (*n =* 5/12) of patients remained as 2 to < 3 mmol/L and 41.7% (*n =* 5/12) decreased to 1 to < 2 mmol/L (data were missing for 2 patients; Fig. 3). In the 16 placebo patients who had a baseline potassium gradient of 2 to < 3 mmol/L, 43.8% (*n =* 7/16) had an increase in gradient to a higher risk category of 3 to < 4 mmol/L, while 43.8% (*n =* 7/16) remained the same (data were missing for 2 patients; Fig. 3).

## Discussion

Recently, Kidney Disease: Improving Global Outcomes (KDIGO) recommended further research determining the efficacy of newer agents in patients with ESKD receiving maintenance hemodialysis [25]. We used data from the phase 3b DIALIZE study to further investigate the spectrum of potassium responses with SZC in maintenance hemodialysis patients with hyperkalemia. SZC was associated with a greater proportion of patients achieving clinically recommended or acceptable pre-dialysis sK^+^ ranges and lower potassium gradient during the evaluation period versus placebo.

Our findings extend those previously reported in the phase 3b DIALIZE study regarding control of sK^+^ with SZC [24]. We assessed control of hyperkalemia using a pre-dialysis sK^+^ range of 4.0–5.0 mmol/L and an extended range of 4.0–5.5 mmol/L. Although UK clinical practice guidelines recommend maintaining a pre-dialysis sK^+^ concentration of 4.0–6.0 mmol/L [26], previous analyses have shown that sK^+^ concentrations of ≥5.6 mmol/L are associated with increased all-cause and cardiovascular mortality [1, 4, 27], and ≥ 5.5 mmol/L is associated with hospitalization [2]. For many physicians and patients, achieving and maintaining a pre-dialysis sK^+^ concentration of 4.0–5.5 mmol/L would be considered a successful response in hemodialysis patients with hyperkalemia, while preventing patients from being exposed to the risks associated with sK^+^ > 5.5 mmol/L. In our analyses, regardless of the sK^+^ range used, SZC was associated with greater control of sK^+^ versus placebo. Using the extended range of 4.0–5.5 mmol/L, nearly half of the patients receiving SZC achieved and maintained the sK^+^ range at all 4 LIDI visits over a 4-week period. Some reduction from baseline in sK^+^ was observed with placebo, which may be attributable to some patients having temporary increases in sK^+^ or to a change in patient behavior such as an increased compliance to diet restrictions.

Reports suggest that a high potassium gradient at the start of hemodialysis is associated with risk of AEs, such as cardiac arrhythmia, mortality, and hospitalization [1, 13–16], probably related to more rapid and larger potassium fluxes during the dialysis session. For instance, Brunelli et al. used a reference potassium gradient of 2 to < 3 mmol/L, and observed an 8, 26, and 59% higher adjusted risk of hospitalization following dialysis with gradient categories of 3 to < 4, 4 to < 5, and ≥ 5 mmol/L, respectively [16]. In addition, the authors observed an increased risk of emergency department visits following dialysis of 6, 17, and 54% with potassium gradient categories of 3 to < 4, 4 to < 5, and ≥ 5 mmol/L, respectively, versus 2 to < 3 mmol/L. Although there was no significant association between risk of mortality and higher potassium gradient, the authors observed a non-significant trend towards a greater risk of cardiovascular hospitalization with ≥5 mmol/L versus 2 to < 3 mmol/L [16]. This led the authors to suggest that the observed associations may be driven by cardiac arrhythmias and their immediate consequences [16]. As a reduction in potassium gradient is often recommended [14, 15], the reduction in potassium gradient to < 3.0 mmol/L with SZC observed in our analyses could potentially lower the risks associated with these factors, although further investigation is required to confirm this.

Practice patterns of dK^+^ concentration are known to vary globally, reflecting the lack of consensus and guidelines on ideal practice. In an analysis of the Dialysis Outcomes and Practice Patterns Study, dK^+^ of 2.0–2.5 mmol/L was most commonly used worldwide, and was prescribed to 75% of patients in the US and > 99% of patients in Japan [4]. In the US, patients are typically prescribed a dK^+^ of 2.0–4.0 mmol/L [4, 16, 28], with approximately 3% of patients receiving 1.0–1.5 mmol/L [4]. Recently, a trend has been observed for steadily increasing dK^+^ concentrations in North America, Europe, Australia, and New Zealand [4], possibly resulting from increased interest in the impact of dK^+^ on hemodialysis outcomes. Several reports have shown that dK^+^ concentrations of 0–1, < 1.5, or < 2 mmol/L are associated with a higher risk of cardiac arrest, sudden death, and all-cause mortality among hemodialysis patients than with higher dK^+^ [10–12]. Furthermore, Karaboyas et al. showed that a dK^+^ concentration of 1.0–1.5 mmol/L is associated with a higher risk of mortality than with 2.0–2.5 mmol/L [4]. Meanwhile, no meaningful differences in clinical outcomes were observed with a dK^+^ concentration of 3.0 versus 2.0 mmol/L [4, 13]. Despite these findings, dK^+^ prescriptions of 1.0–1.5 mmol/L or lower are still used; e.g. 1.0–1.5 mmol/L is used by 62% of patients in Spain and 9% of patients in the Gulf Cooperation Council [4]. Indeed, dK^+^ concentration should be no lower than is necessary to achieve potassium control, and recent publications have recommended avoiding dK^+^ of < 2.0 mmol/L [4, 12, 28–31].

Approaches other than modifying hemodialysis factors, such as pharmacological options and education on dietary potassium sources, may merit further attention to improve hemodialysis outcomes [4, 13, 16, 28]. Concentrations of sK^+^ are typically monitored once a month in clinical practice, but more frequent monitoring of pre-dialysis sK^+^ may allow appropriate adjustment of dK^+^ concentration [1, 12, 31]. In DIALIZE, dK^+^ modification was based on investigator’s choice and locally accepted clinical practice [24]. As demonstrated in the present analyses, the potential beneficial effect on potassium gradient is due to the reduction in pre-dialysis sK^+^ with SZC, since so few patients had an increase in dK^+^ during the study. In the long-term, this may result in the ability to relax dietary potassium restrictions allowing a diet that is richer in nutrients and fiber, as well as a potential reduction in additional acute dialysis treatments, which could improve patients’ quality of life; however, this requires further investigation.

The present analyses have several limitations. Although the analyses provide interesting findings requiring further study, they are post-hoc in nature and were not prespecified; therefore, the results are exploratory and hypothesis-generating. Patient numbers within the potassium gradient categories were small, limiting the interpretation of these findings. Target sK^+^ varies in clinical practice according to local guidelines; as such, the ranges analyzed may not be applicable worldwide. The changes in potassium gradient seen were predominantly due to reduction in pre-dialysis sK^+^ with SZC, and so the potential benefit of increasing dK^+^ baths could not be explored. Finally, clinical characteristics at baseline that may contribute to control of hyperkalemia or reduction in potassium gradient were not explored. However, the analyses presented here have several strengths. Specifically, the analyses are derived from a randomized controlled trial with a robust methodology that included blinding, multiple study centers, and repeated measures during several LIDI visits.

## Conclusions

In conclusion, these analyses expand our knowledge of the spectrum of potassium response with SZC in maintenance hemodialysis patients with hyperkalemia. Our findings suggest that treatment with SZC improves control of hyperkalemia in maintenance hemodialysis patients with hyperkalemia. A reduction in potassium gradient towards values below the reported higher risk of > 3.0 mmol/L with SZC was observed largely without changing dK^+^. Further investigation is required to determine whether this finding could potentially modify the risks associated with a high potassium gradient.

## Data Availability

These post-hoc analyses were derived from the DIALIZE study (NCT03303521); the full details have been presented previously [24]. Data underlying the findings described in this manuscript may be requested in accordance with AstraZeneca’s data sharing policy described at https://astrazenecagroup-dt.pharmacm.com/DT/Home by accessing www.vivli.org.

## Abbreviations

AE: Adverse event
CI: Confidence interval
dK^+^: Dialysate potassium
EOT: End of treatment
ESKD: End-stage kidney disease
KDIGO: Kidney Disease: Improving Global Outcomes
LIDI: Long interdialytic interval
SD: Standard deviation
SIDI: Short interdialytic interval
sK^+^: Serum potassium
SZC: Sodium zirconium cyclosilicate
